# Diffuse reflectance spectroscopy for breach detection during pedicle screw placement: a first in vivo investigation in a porcine model

**DOI:** 10.1186/s12938-020-00791-2

**Published:** 2020-06-12

**Authors:** Akash Swamy, Jarich W. Spliethoff, Gustav Burström, Drazenko Babic, Christian Reich, Joanneke Groen, Erik Edström, Adrian Elmi-Terander, John M. Racadio, Jenny Dankelman, Benno H. W. Hendriks

**Affiliations:** 1 Department of Biomechanical Engineering, Delft University of Technology, Mekelweg 2, 2628 CD Delft, The Netherlands; 2 Department of In-body Systems, Philips Research, Royal Philips NV, High Tech Campus 34, 5656 AE Eindhoven, The Netherlands; 3 Department of Clinical Neuroscience, Karolinska Institutet, Stockholm, Sweden; 4 Department of Neurosurgery, Karolinska University Hospital, Stockholm, Sweden; 5 Cincinnati Children’s Hospital Medical Center, 3333 Burnet Avenue, Cincinnati, OH USA

**Keywords:** Diffuse reflectance spectroscopy, In vivo, Spinal screw placement, Spine

## Abstract

**Background:**

The safe and accurate placement of pedicle screws remains a critical step in open and minimally invasive spine surgery, emphasizing the need for intraoperative guidance techniques. Diffuse reflectance spectroscopy (DRS) is an optical sensing technology that may provide intraoperative guidance in pedicle screw placement.

**Purpose:**

The study presents the first in vivo minimally invasive procedure using DRS sensing at the tip of a Jamshidi needle with an integrated optical K-wire. We investigate the effect of tissue perfusion and probe-handling conditions on the reliability of fat fraction measurements for breach detection in vivo.

**Methods:**

A Jamshidi needle with an integrated fiber-optic K-wire was gradually inserted into the vertebrae under intraoperative image guidance. The fiber-optic K-wire consisted of two optical fibers with a fiber-to-fiber distance of 1.024 mm. DRS spectra in the wavelength range of 450 to 1600 nm were acquired at several positions along the path inside the vertebrae. Probe-handling conditions were varied by changing the amount of pressure exerted on the probe within the vertebrae. Continuous spectra were recorded as the probe was placed in the center of the vertebral body while the porcine specimen was sacrificed via a lethal injection.

**Results:**

A typical insertion of the fiber-optic K-wire showed a drop in fat fraction during an anterior breach as the probe transitioned from cancellous to cortical bone. Fat fraction measurements were found to be similar irrespective of the amount of pressure exerted on the probe (*p* = 0.65). The 95% confidence interval of fat fraction determination was found in the narrow range of 1.5–3.6% under various probe-handling conditions. The fat fraction measurements remained stable during 70 min of decreased blood flow after the animal was sacrificed.

**Discussions:**

These findings indicate that changes in tissue perfusion and probe-handling conditions have a relatively low measureable effect on the DRS signal quality and thereby on the determination of fat fraction as a breach detection signal.

**Conclusions:**

Fat fraction quantification for intraoperative pedicle screw breach detection is reliable, irrespective of changes in tissue perfusion and probe-handling conditions.

## Background

In spinal fusion surgery, pedicle screws are placed into the vertebrae and connected with rods to fuse parts of the spine in order to regain and maintain spinal stability [1]. Serious vascular and neurological injuries can occur due to inaccurate placement of pedicle screws [2–4]. Currently, there is a trend toward minimally invasive surgery (MIS) [5], owing to benefits such as reduced surgical trauma, decreased postoperative pain and shortened hospital stays [6]. However, MIS requires technical aids, since the small surgical wounds do not allow visualization of anatomical landmarks. Several guidance techniques including navigation systems and other non-imaging-based techniques have been developed for safe and accurate pedicle screw placement [7]. However, accuracy rates of pedicle screw placement reported in the literature vary widely. Meta-analysis studies indicate that using the free-hand technique, breaches of greater than 4 mm occur in 1–6.5% of the placed pedicle screws [8–10]. Based on a recent systematic review by Staartjes et al. [11], these inaccuracies lead to screw revisions in 6.0% of operated patients. Misplaced screws can also lead to new complications and extended hospital stays [12]. Therefore, using pedicle screw guidance solutions may further improve the safety of these procedures.

Currently, surgeons may use an electrical conductivity-based device to assist them in detecting impending breach [13, 14]. However, this device is known to be affected by variations in probe-handling conditions during the maneuvering of the probe in perfused vertebrae [15]. Neurophysiological monitoring techniques, whereby a placed pedicle screw is electrically stimulated and distal motor responses are monitored, can also be used for breach detection but are employed only after the screws are placed and are known to have low sensitivity in identifying screw misplacement [16]. Thus, this technology cannot serve to predict or prevent a breach.

Real-time monitoring of tissue characteristics using diffuse reflectance spectroscopy (DRS) at the tip of an instrument may offer a new possibility for intraoperative guidance. The technology can be used to determine physiological parameters such as fat and water content and fat fraction, within the tissues ahead of an instrument and has previously been investigated for detection of breaches during pedicle screw placement [17, 18]. Using a custom-built screw probe with integrated optical fibers to sample fat fraction within vertebrae, the technology could accurately predict the transition from cancellous to cortical bone with high sensitivity and specificity [18]. However, these studies were all performed ex vivo and the possible influence of blood at the tip of the probe could not be studied.

Thus, a natural next step is to gauge the reliability of the DRS technique in an in vivo setting. Spliethoff et al. [19] investigated the clinical use of DRS for lung biopsy guidance in an in vivo setting and found that the reliability of DRS measurements was not influenced by the presence of blood due to tissue perfusion. However, the findings from lung biopsy cannot be assumed to be transferrable to the case of breach detection in the spine. The tissue composition and the effects of perfusion within the vertebrae need to be fully understood to develop a novel and robust breach detection algorithm. Moreover, the effect of changes in probe-handling or probe contact pressure on the in vivo DRS measurements needs to be studied. Literature shows that the spectral response to probe contact pressure is complex, and dependent upon several factors including probe contact area, wavelength window, tissue type and the probe operator involved. The majority of these studies focused on application of probe pressure on soft tissues such as human skin, liver and heart [20–24]. However, the effect of contact pressure on DRS-based physiological measurements in vertebrae has not been studied. Therefore, the purpose of this study was to investigate the reliability of fat fraction measurements as a breach detection method, in an in vivo model during different probe-handling conditions and during normal as well as abnormal tissue perfusion conditions.

## Results

**Fig. 1:**
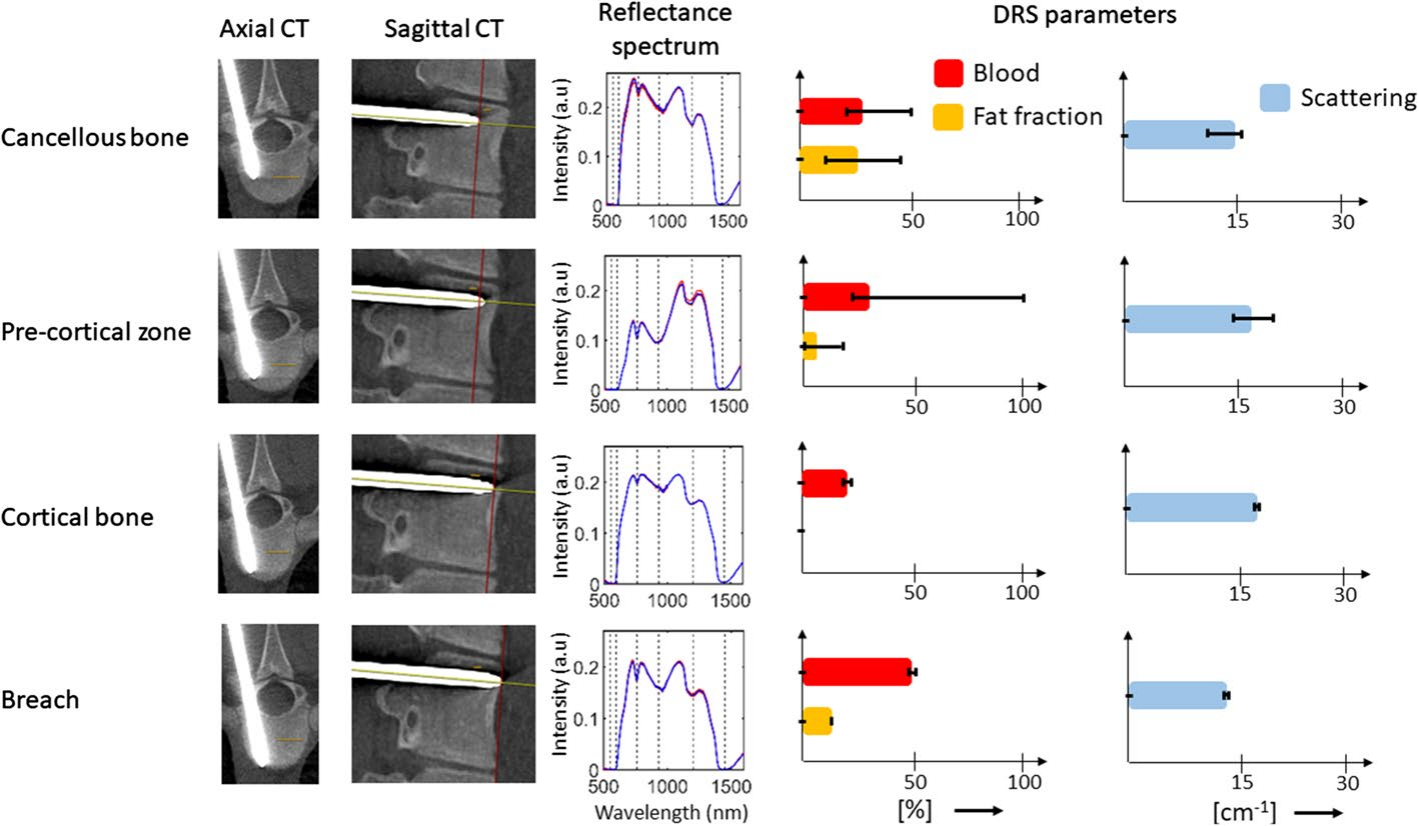
Example of an insertion into a porcine vertebra in vivo. DRS readings and associated imaging of an anterior fiber-optic K-wire breach. First and second columns depict axial and sagittal computed tomographies of each position, respectively. The third column depicts the measured spectra at each position in red and corresponding fitted spectra in blue. The fourth column shows the median values of blood content and fat fraction with error bars indicating min and max values. The fifth column represents the variation of reduced scattering amplitude at 800 nm

An illustration of an anterior breach is presented in Fig. 1. The initial position of the fiber-optic K-wire in cancellous bone shows a median blood content of 28.9% [min 21.4–max 51.3] and median fat fraction of 26.4% [min 11.9–max 50.0]. The transition of the fiber-optic K-wire from cancellous bone to Pre-cortical zone (PCZ) led to a drop in fat fraction to 5.3% [min 0–max 17.3], while the median blood content remained at 28.8%. However, the blood content in the PCZ showed high variability (min 21.0%–max 100%). As the fiber-optic K-wire progressed into the cortical bone, the median blood content declined to 19.4% [min 18.9–max 20.5] and median fat fraction dropped to 0% [min 0–max 0.1]. Finally, as the fiber-optic K-wire breached the cortical bone boundary, both the median blood content, 48.9% [min 48.1–max 49.6] and median fat fraction, 11.2% [min 10.9–max 11.4] showed a sharp increase, respectively. The median scattering amplitude during the insertion ranged between 14.6 and 17.2 cm^−1^.

**Fig. 2:**
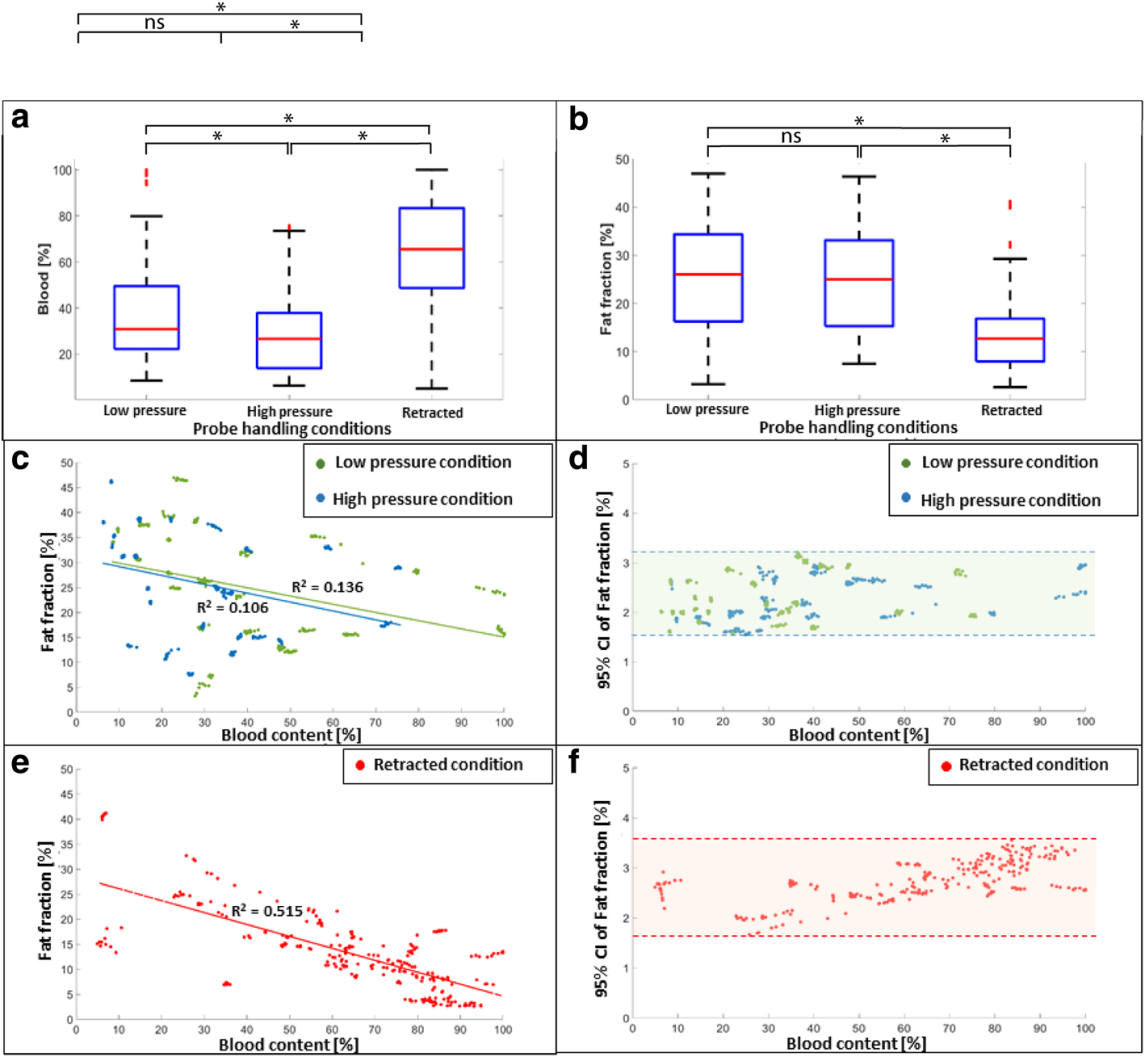
Effect of probe-handling conditions on DRS-based physiological parameters. **a**, **b** Box plots depicting effect of probe-handling conditions on blood and fat fraction. **c**, **e** Scatter plot depicting distribution of blood and fat fraction due to various probe handing conditions. **d**, **f** 95% confidence interval of fat fraction determination due to various probe-handling conditions

The amount of locally sampled blood content is found to be significantly higher during retracted probe condition as compared to low- and high-pressure probe conditions (*p* < 0.01). This blood content was also found to differ between low- and high-pressure probe conditions (*p* < 0.01) as shown in Fig. 2a.

Meanwhile, the amount of locally sampled fat fraction in front of the probe tip was found to be constant during low- and high-pressure probe conditions (*p* = 0.65), as shown in Fig. 2b. Whereas, the amount of fat fraction was found to be significantly lower during retracted probe condition as compared to both low- and high-pressure probe conditions (*p* < 0.01).

Blood content and fat fraction estimated for low- and high-pressure probe-handling conditions have a limited relationship between each other as indicated by the low regression values in Fig. 2c. Nevertheless, the range of 95% confidence interval [1.5–3.2%] indicates a high confidence of the estimation of fat fraction values for low- and high-pressure probe conditions from the DRS spectra as depicted in Fig. 2d.

However, the retracted probe condition does indicate a trend toward a decrease in fat fraction as the locally probed blood content increases (*R*^2^ = 0.515) as shown in Fig. 2e. The narrow 95% confidence interval range [1.7–3.6%] indicates a high reliability of estimated values from the DRS spectra (Fig. 2f).

**Fig. 3:**
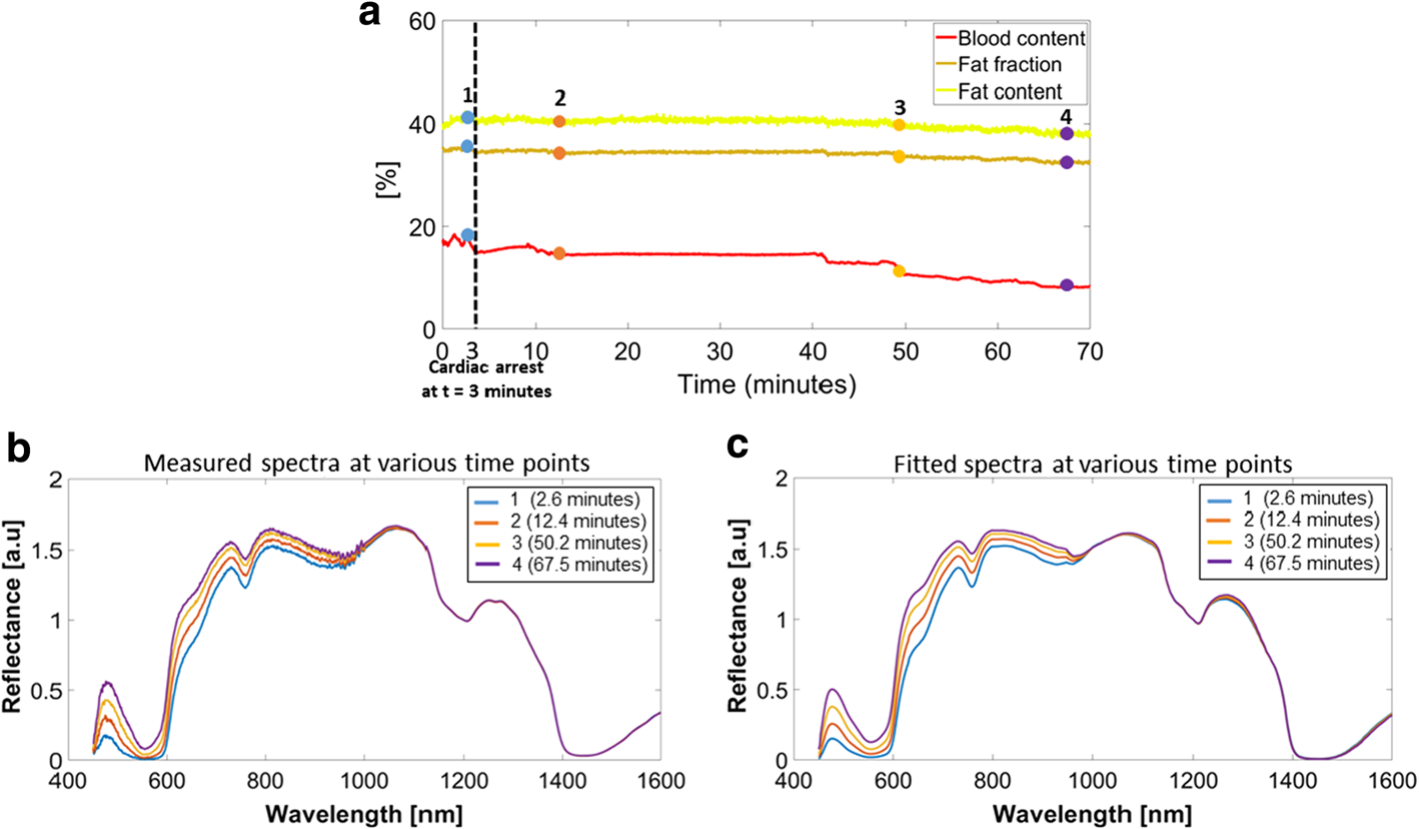
**a** Temporal changes in blood, fat fraction and fat during animal killing. **b**, **c** Normalized spectra at 1200 nm shown at various time points for measured and fitted spectra, respectively

Blood content in the vertebral body decreased from 18.9 to 8.1% during 70 min from the time of cardiac arrest of the animal as depicted in Fig. 3a. However, the fat fraction and fat content remained relatively stable with a decrease from 35.5 to 32.3% and from 41.5 to 37.7%, respectively.

## Discussion

In the present study, we investigated the reliability of fat fraction measurements as a breach detection signal during different tissue perfusion and probe handing conditions using a fiber-optic K-wire inserted in porcine vertebrae in a minimally invasive in vivo setting. Optical fibers integrated into a K-wire were inserted into the vertebrae under image guidance while DRS measurements were recorded at various positions of the probe. The results of this study confirm that a breach can be detected in an in vivo setting. Sufficient contact between probe and perfused vertebral tissue ensures reliable DRS measurements. Moreover, changes in probe-handling conditions had a minor impact on the quality of the DRS measurements. Continuous DRS measurements during sacrifice of the animal indicated that the main discriminating parameter for breach detection, namely fat fraction, is largely unsusceptible to changes in tissue perfusion.

A fiber-optic K-wire approaching the cortical bone boundary showed a sharp drop in DRS-based fat fraction during the transition of the probe from cancellous bone to PCZ and cortical bone (Fig. 1). This finding agrees with previous publications on human cadavers [17, 18]. However, a high variability of blood content and fat fraction in cancellous bone and PCZ regions within the vertebra was found as represented by the error bars in Fig. 1. A possible reason for such variation in the locally sampled blood and fat fraction can be attributed to the heterogeneity within the tissue due to the random distribution of vascular structures and triglyceride storage sites [25–27]. This finding is supported by the fact that cancellous bone is known to house red and yellow bone marrow within its organic matrix for erythropoiesis and fat storage, respectively [28]. Another possible cause for the high variability in blood content can be attributed to the filling of the bone cavity from a prior retracted probe measurement condition, influencing subsequent measurements.

The relatively low blood content in cortical bone as compared to cancellous bone is in correspondence with previously published data [26, 27].

Another important aspect investigated was the effect of tissue perfusion on the fat fraction and blood content measurements due to varying probe-handling conditions.

A significantly higher blood content was measured when the probe was retracted within the vertebrae (Fig. 2a). This finding makes intuitive sense since a void at the tip of the probe is expected to be created and blood tends to flow into the void space due to the temporary retraction of the probe. The fact that fat fraction significantly drops during the retracted probe condition further strengthens this claim as the composition of this bony void would mainly be a blood-filled pool (Fig. 2b, e). The implication of the dependence of blood and fat fraction values on retracted handling conditions is clinically relevant since it provides an indication of the directionality of the probe within the vertebrae. Since a high blood content and low fat fraction measurement most likely indicate that the probe has been retracted. Such information, derived from quantifying blood content primarily from the visible wavelength range of 450–600 nm, can be important for the surgeon for intraoperative guidance of the fiber-optic K-wire during pedicle screw placement.

Lower blood content was measured when high pressure was exerted as compared to low probe pressure. This finding is in line with previously published studies which show an inverse dependence of probe contact pressure and local hemodynamics due to the decrease in local blood volume under focal pressure [20, 24]. Similar fat fraction measurements were observed during low- and high-pressure probe conditions (*p* = 0.65) indicating that manual probe-handling changes (i.e., using manual pressure, hammer or drill) do not affect the fat fraction measurements as long as there is a direct contact between the probe tip and the bone surface (Fig. 2b). Cugmas et al. [20] observed an inverse dependence of exerted probe pressure and water content in the 950–1600 nm wavelength window. In the present study, the effect on fat and water content was minor. The main cause of discrepancy may be attributed to the relatively lower probe pressure-induced local deformation of vertebral bone, due to its higher mechanical strength (Young’s modulus), as compared to human skin.

The estimation of signal quality or the signal to noise ratio of the spectra recorded during varying probe-handling conditions was also important to quantify in order to avoid spurious fat fraction measurements. The 95% confidence intervals of determining fat fraction varied in a relatively narrow range of 1.5–3.6% under three probe-handling conditions (Fig. 2d, f). This finding implies that tissue perfusion does not significantly affect the quality of the DRS spectra. Thus, the fat fraction determined from the fitting model can be considered to reliably reflect the local tissue composition despite the inevitable presence of blood around the probe tip.

The fact that the fat fraction signal remained almost unaffected during decreased blood circulation within the porcine specimen further points toward the reliability of fat fraction as a breach detection signal (Fig. 3). This finding also further validates previous cadaveric studies which investigated the use of intraoperative fat fraction measurements to detect breaches during pedicle screw placement procedures [17, 18, 25]. The stable nature of the fat fraction signal during continuous DRS measurement within the vertebral body, 70 min after the time of cardiac arrest, indicates the relative independence of fat and fat fraction measurements as compared to blood content measurements. After cardiac arrest, DRS measurements show a gradual decrease in vertebral blood content (time points 1–4, Fig. 3a). In line with this, the measured reflectance spectral intensity increases in the wavelength range of 530–580 nm (Fig. 3b), the wavelength band known to be the region of maximum absorptivity of blood chromophore derivatives [29]. This phenomenon may be explained by the gradual settling of blood, livor mortis, once circulation ceases [30]. However, it is known that the fat and water chromophores are mainly sensitive to light in the near-infrared wavelength range between 1000 and 2200 nm [31]. Figure 3b indicates that the normalized reflectance intensity in the range of 1000–1600 nm for various time points showed a complete overlap after normalizing the spectra around the wavelength of 1200 nm. Figure 3c depicting the reflectance spectra derived from the fitting model shows a similar trend. Thus, confirming that DRS determined blood and fat fraction values independently reflect the locally probed tissue composition.

The noise within the fat and fat fraction signals in Fig. 3a can be attributed to the assumptions made in the fitting model.

It must be noted that the blood content defined in this study was based on the average hemoglobin concentration of a normal human. It is known that the average hemoglobin concentration of a pig is lower [32]. However, this aspect is expected to have a minor effect on the findings since relative trends rather than absolute values are of interest.

The configuration of the fiber-optic K-wire allows integration into different surgical instruments. For example, it can be integrated into a manual pedicle probe (gear shift) or into a drill for quick and safe pedicle cannulation and K-wire placement.

Previously, an electrical conductivity-based device has been investigated as a breach detection tool [33]. It has the drawback that the quality of the feedback is susceptible to probe-handling conditions [15]. The method of breach detection applied in this study presents the possibility of measuring the local blood content and fat fraction. A combination of those has the potential to serve as a reliable feedback during pedicle screw placement procedures. However, further investigations are required to verify this claim.

## Conclusion

This study investigated the reliability of fat fraction measurement as a breach detection method during tissue perfusion and different probe-handling conditions. We have demonstrated that fat fraction quantification for intraoperative pedicle screw breach detection is reliable, irrespective of changes in tissue perfusion and probe-handling conditions.

## Methods

### Porcine animal model

An animal experiment was conducted at the Cincinnati Children’s Hospital Medical Center, Ohio, United States. The study was approved by the Institutional Animal Care and Use Committee in compliance with the ethical guidelines for animal studies. For the experiment a 5-month and 18-day-old pig weighing 78.4 kg was used in an in vivo setting under general anesthesia. The animal was euthanized at the conclusion of the experiment. Additional data were collected during and after this phase.

### Experimental design

**Fig. 4:**
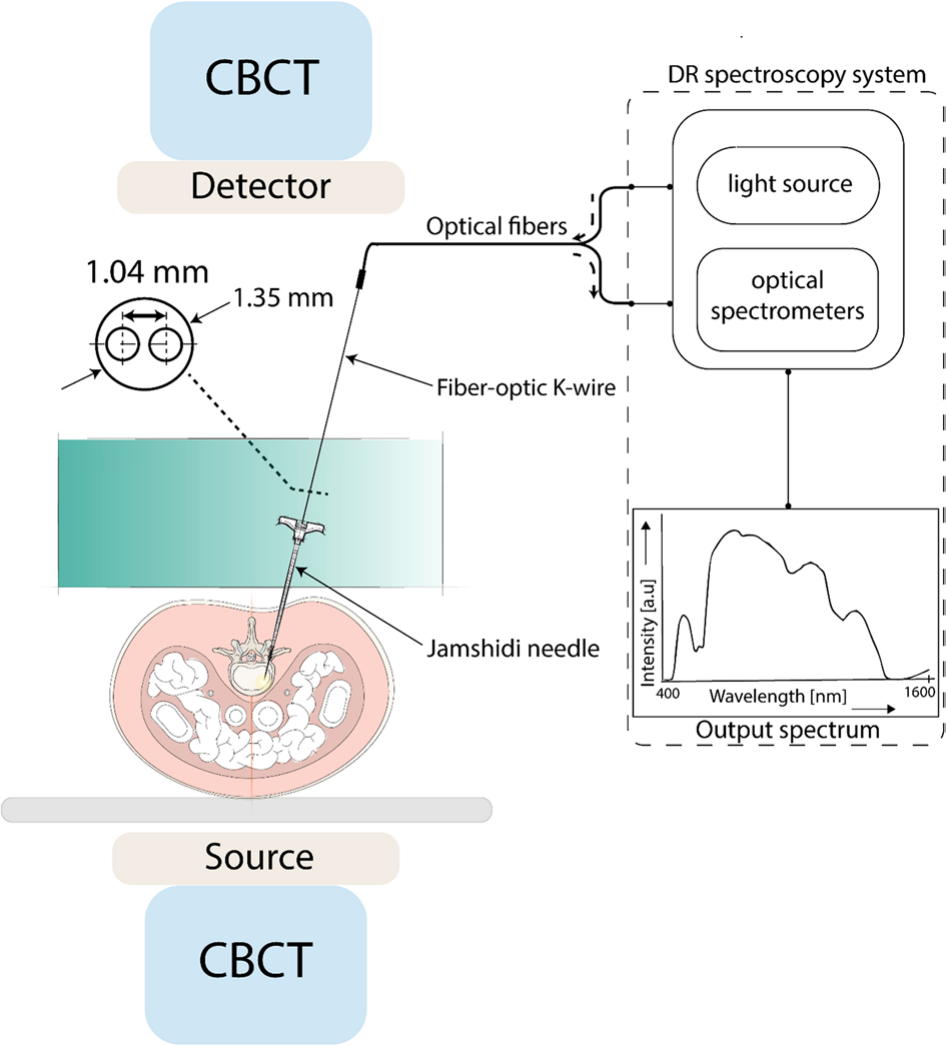
Schematic representation of the experimental setup

The animal was positioned on the table in prone position on an operating table. Cone beam computed tomography (CBCT) images were acquired (AlluraClarity FD20; Philips Healthcare, Best, the Netherlands) and an augmented reality surgical navigation system (ARSN) was used to plan the trajectories of the insertions [34]. After an initial skin incision, a Jamshidi needle was navigated toward the pedicle with ARSN guidance as previously described [35, 36]. Once the entry point on the vertebrae was encountered, the Jamshidi needle was hammered, in a stepwise fashion to penetrate the pedicle and the vertebral body. The position of the Jamshidi needle along with the navigation path was verified by acquiring consecutive CBCT images. Once bone purchase of the Jamshidi needle within the vertebra was achieved, the inner stylet was removed and the fiber-optic K-wire was introduced to obtain DRS measurements as shown in Fig. 4. A set containing three types of measurement conditions was performed for each position: (first) 10 DRS measurements were acquired while a low axial pressure was exerted to the probe (1.2 ± 0.3 N/mm^2^); (second) 10 measurements were acquired while a high axial pressure was exerted (6.1 ± 0.5 N/mm^2^); (third) the fiber-optic K-wire was retracted by 1–2 mm from its initial position and 10 measurements were performed. Low and high probe pressures were quantified in a prior study. The fiber-optic K-wire was then removed, the inner stylet reinserted, and the Jamshidi needle was then hammered to a new position along the intended trajectory. Additional CBCT’s were acquired whenever a change in DRS readings was encountered to document positions along an insertion trajectory.

A final analysis of the effect of blood flow and tissue perfusion, on DRS parameters, was performed once all other measurements were taken; the porcine specimen was sacrificed by a lethal injection while the fiber-optic K-wire was left to record continuously (70 min) in the center of a vertebral body.

### DRS instrument

The vertebrae were probed using a Jamshidi needle with a K-wire integrated with two optical fibers at the tip of the probe, i.e., the fiber-optic K-wire (diameter 1.35 mm). The fiber-to fiber distance was 1.042 mm (Fig. 4). The fiber distance was chosen based on experience from previous studies and is related to the largest light penetration depth achievable for high-quality DRS measurements and to ensure mechanical durability needed for bone sampling [17]. One fiber was connected to a tungsten halogen broadband light source (Avantes AvaLight-Hal-S) to transmit the light into the tissue, while the second, receiving fiber, was connected to two optical spectrometers (via a fiber splitter) as shown in Fig. 4. The core diameter of the source and receiving fibers was 200 µm. The spectrometers resolved the light in the visible (Maya2000 Pro, Ocean Optics) and near-infrared wavelength regions (NirQuest 512, Ocean Optics), to produce a spectrum in the wavelength range of 450–1600 nm. An in-house developed LabVIEW software (National Instruments, Austin, Texas) was used to control the spectrometers and perform data acquisition. The general principles of the DRS system along with the calibration method have been described previously [37–39].

### Data analysis

Six insertions were performed in six thoracic and lumbar vertebrae. A total of 270 spectra were recorded in vivo and analyzed further. Continuous DRS measurements were performed while the specimen was killed, and a total of 2020 spectra were recorded for further analyses.

Tissue labeling of DRS measurements were performed by a trained physician, blinded to the DRS spectral readings, based on anatomical position of the probe on the CBCT verification scans. Anatomical regions of interest were defined as cancellous bone, cortical bone, pre-cortical zone (PCZ) and breach based on experience from a previous study [18]. PCZ was defined as the distance within 3 mm from cortical bone boundary. Breach was defined as the first 3 mm outside the vertebrae after the probe broke through the cortical bone [18].

### Determination of DRS-based physiological parameters

A previously described fitting model was used to translate the measured diffuse reflectance spectra into meaningful physiological parameters [38–40]. The model estimates the absorption *µ*_a_ (*λ*) and reduced scattering coefficient *µ*_sʹ_ (*λ*) expressed in cm^− 1^. From the a priori knowledge of fiber distance and wavelength-dependent absorption coefficients, the amount of deoxygenated hemoglobin (Hb) and oxygenated hemoglobin (HbO_2_), fat and water present in the probed tissue was determined using a previously described method [37]. The reduced scattering amplitude at a normalization wavelength of 800 nm was also calculated [38]. The blood content is defined as the total concentration of hemoglobin which is the sum of concentrations of oxyhemoglobin and deoxyhemoglobin. It is expressed as a percentage of total concentration of hemoglobin in normal human blood (150 g/l). The fat fraction is defined as a function of fat and water content, as previously described [31] and calculated as follows:

$${\text{Fat}}\;{\text{fraction}}\, \left[ \% \right] = \frac{\text{Fat}}{{{\text{Fat}} + {\text{Water}}}} \times 100.$$

### Statistical analysis

A Jarque–Bera test was used to test each dataset for normality. All datasets were found not to be normally distributed. Therefore, the estimated physiological parameters were calculated as median values and the variation was represented as minimum and maximum values. A nonparametric Wilcoxon rank-sum test was used to perform inferential statistics, to compare the effects of probe pressure on DRS determined blood and fat fraction datasets. Confidence intervals of the physiological parameters derived from the fitting model were calculated to estimate the statistical errors of the fit parameters according to the method described previously by Amelink et al. [41]. Thus, the confidence interval range provides an indication of the signal to noise ratio and thereby the quality of the DRS measurements. Normalization of the spectra was applied at 1200 nm, to qualitatively compare spectral recordings during animal sacrifice. The significance value was set to 0.01.

## Data Availability

The datasets used and/or analyzed during the current study are available from the corresponding author on reasonable request.

## Abbreviations

DRS: Diffuse reflectance spectroscopy
MIS: Minimally invasive surgery
PCZ: Pre-cortical zone
CBCT: Cone beam computed tomography
